# The Relationship Between Simple Renal Cysts and Renal Function in Patients With Type 2 Diabetes

**DOI:** 10.3389/fphys.2020.616167

**Published:** 2020-12-15

**Authors:** Ling Wei, Ying Xiao, Xiaofen Xiong, Li Li, Yuan Yang, Yachun Han, Hao Zhao, Ming Yang, Lin Sun

**Affiliations:** Hunan Key Laboratory of Kidney Disease and Blood Purification, Department of Nephrology, The Second Xiangya Hospital, Central South University, Changsha, China

**Keywords:** simple renal cysts, type 2 diabetes, renal function, proteinuria, estimated glomerular filtration rate

## Abstract

**Introduction**: Simple renal cysts (SRCs) are the most common acquired cystic kidney disease, but the relationship between SRCs and renal function has not been clarified in patients with type 2 diabetes mellitus (T2DM).

**Methods**: A retrospective study was conducted to analyze the clinical features of renal cysts and ultrasound data of the kidney in 4,304 patients with T2DM.

**Results**: The prevalence of SRCs in patients with T2DM was 21.1%. Compared to patients with no SRCs, patients with SRCs had worse renal function (estimated glomerular filtration rate: 108.65 ± 40.93 vs. 92.38 ± 42.1 ml/min/1.73 m^2^, *p* < 0.05). After adjusting the confounders, SRC was related to estimated glomerular filtration rate in patients with T2DM [odds ratio = 1.49, 95% confidence interval (1.24, 1.79), *p* < 0.01]. Age, gout, proteinuria, cerebrovascular disease (CVD), and increased serum phosphorus levels were associated with SRCs in patients with T2DM.

**Conclusion**: SRCs are associated with worse renal function in patients with T2DM. More attention should be paid to gout, proteinuria, CVD, serum phosphorus levels, and renal function in T2DM patients with SRCs.

## Introduction

Simple renal cysts (SRCs) are the most common acquired cystic kidney disease. Microdissection suggests that they might be the diverticula of the distal convoluted tubules or collecting ducts (Baert and Steg, 1977). The incidence of SRCs is 2.38% in the second decade of life and 35.8% in the seventh or greater decades of life in healthy people (Chang et al., 2007). SRCs have been associated only with hypertension and not renal insufficiency in other studies (Chin et al., 2006; Ozdemir and Kapucu, 2017). However, according to Qiu et al. (2017), the presence of SRCs in donor kidneys potentially alters early and long-term allograft function. Currently, the risk factors for SRCs remain to be elucidated.

According to a recent study, approximately 463 million patients had been diagnosed with diabetes through 2019, of which type 2 diabetes mellitus (T2DM) accounting for around 90%. The number is increasing annually (International Diabetes Federation, 2019). The associations between SRCs and diabetes, including diabetic nephropathy (DN), remain unclear. In some studies, no association was observed between the incidence of diabetes and renal cysts, although hypertension, hyperlipidaemia, and obesity were more common in patients with SRCs than in patients without renal cysts (Suher et al., 2006; Choi, 2016). However, several other studies indicated that diabetes or hyperglycaemia was more common in patients with renal cysts (Mosharafa, 2008; Ozveren et al., 2016). Therefore, the relationship between diabetes and renal cysts remains controversial. Further studies are needed to determine whether the presence of renal cysts may indicate an early decrease in renal function in patients with T2DM. Age, body mass index (BMI), proteinuria, microscopic haematuria, estimated glomerular filtration rate (eGFR), and hypertension have been reported to be independent risk factors for SRCs in healthy subjects. Few studies have examined the relationship between SRCs and renal function, as well as related factors for SRCs, in patients with T2DM.

The study aimed to demonstrate the relationship between renal cysts and renal function and analyze the related factors for SRCs in patients with T2DM.

## Materials and Methods

### Subjects

Seven thousand three hundred ninety-eight patients with T2DM who were hospitalized in the Department of Endocrinology in the Second Xiangya Hospital of Central South University in Hunan Province, Changsha City between 2014 and 2018 were enrolled. The inclusion criteria were (i) patients diagnosed with T2DM according to the criteria of the World Health Organization published in 1999 (Alberti and Zimmet, 1998) and (ii) patients who underwent renal and ureteral ultrasonography. The exclusion criteria were (i) patients who were younger than 18 years; (ii) patients with malignant tumors, blood disease, polycystic kidney disease, acute kidney injury, and other kidney diseases; (iii) patients who were pregnant; and (iv) patients with missing data. Ultimately, 4,304 patients were included in the study. The study was performed according to the principles of the Declaration of Helsinki. The requirement for individual consent was waived for this retrospective study. This study was approved by the Medical Ethics Committee of the Second Xiangya Hospital of Central South University [(2019) Ethics review division no. 074].

### Data Collection

Gender, age, disease duration, family history, concomitant disease, the current therapy, disease course, smoking history, drinking history, BMI [BMI = weight (kg)/height^2^ (m^2^)], and waist-hip ratio were all obtained from the medical records of each patient. Venous blood samples were collected in the morning after an overnight fast to measure the serum concentrations of total cholesterol (TC), total triglycerides (TG), high-density lipoprotein cholesterol (HDL-C), low-density lipoprotein cholesterol (LDL-C), albumin (ALB), fasting plasma glucose (FPG), glycosylated hemoglobin (Hb1Ac), uric acid (UA), serum creatinine (SCr), phosphorus, and calcium. The plasma creatinine in our hospital was measured by Jaffe’s kinetic method on a Hitachi analyzer. The eGFR (ml/min/1.73 m^2^) was calculated by using the recommended modified glomerular filtration rate estimating equation for Chinese patients: 175 × [serum creatinine concentration (μmol/L)/88.4]^−1.234^ × [age (years)]^−0.179^ × (0.79 if female) (Ma et al., 2006). The ultrasound was conducted by one experienced sonographer in the ultrasound department of our hospital. If the results were in dispute, another professional sonographer would participate in the discussion to reach a conclusion. And SRCs were defined as round or elliptical in shape, with a thin, smooth outer wall, posterior enhancement, and no internal debris or septum. The length and width of the largest renal cyst were collected. The size of the SRCs was measured by the cross-sectional area of the largest renal cyst [S = the length (mm) × width of the largest renal cyst (mm)]. Accompanying diseases were collected from medical records of patients, including diabetic retinopathy (DR), peripheral vascular disease (PVD), diabetic autonomic neuropathy (DAN), diabetic foot (DF), DN, diabetic ketosis (DK), coronary heart disease (CHD), cerebrovascular disease (CVD), and urinary tract infection (UTI). The therapies of patients during the hospital were collected from their medical records including the use of angiotensin converting enzyme inhibitors (ACEIs), angiotensin receptor blockers (ARBs), lipid-lowering therapy, oral hypoglycemic agent, and insulin therapy.

### Statistical Analyses

The study data were analyzed using SPSS version 25.0 and GraphPad Prism version 8 software for Windows. Normally distributed continuous variables are presented as the means ± SD. Continuous variables with a skewed distribution are reported as medians (interquartile ranges 25–75%). The normal distribution of all continuous variables was evaluated using a graphical method. Categorical variables are reported as the number and percentage of patients in each group. The relationship between the normally distributed continuous variables and renal cysts was statistically analyzed using Student’s *t*-test, the skewed continuous variables were statistically analyzed using the Mann-Whitney U test, and the distributed variables were compared using the Chi-square test in the analysis of the baseline characteristics. Continuous variables with statistically significant differences (*p* < 0.05) in the Student’s *t*-test were converted into categorical variables. Regarding the conversion criteria, age was grouped based on the median age of 59 years old, systolic blood pressure (SBP) was grouped based on 140 mmHg, the duration of the disease was grouped into less than 10 years and more than 10 years, and waist-to-hip ratio was grouped based on 0.9 in males and 0.8 in females. BMI was divided into three groups including less than 24 kg/m^2^, 24~28 kg/m^2^, and more than 28 kg/m^2^. Hemoglobin, PLT, TG, TC, FPG, HbA1c, SCr, UA, urine PH, and the concentration of serum phosphorus and calcium were divided by the normal value or upper and lower limit value, a 24 h urinary albumin level <30 mg/d was defined as the no albuminuria group and ≥30 mg/d was divided into microalbuminuria (MIAU) or macroalbuminuria (MAAU) group. Second, multivariable logistic regression analysis was conducted to estimate the associations between the variables and eGFR in patients with T2DM as described previously (Xiong et al., 2017). Briefly, the variables that was statistically significant differences in the univariables analysis were included after excluding the relevant variables with a large correlation. The variables selected were gender, age, duration, smoking, drinking, BMI, family history, urolithiasis, hemoglobin, TC, TG, LDL, ALB, HbA1c, UA, 24 h urinary albumin, calcium, phosphorus, DR, DPN, DAN, DF, DN, DK, hypertension, CVD, CHD, fatty liver, UTI, ACEIs, ARBs, lipid-lowering therapy, oral hypoglycemic agent, insulin therapy, and SRC.

In addition, multivariable logistic analysis was also used for estimating the association of variables and SRCs. Variables that were significantly different between patients with and without SRCs were included in the logistic analysis, including age, gender, disease duration, waist-hip ratio, hemoglobin, PLT, TG, TC, FPG, HbA1c, SCr, 24 h urinary albumin, calcium, phosphorus, smoking, drinking, urolithiasis, history of gout, DF, DK, hypertension, CVD, fatty liver, and insulin therapy. A multivariate logistic regression analysis was conducted to identify the risk factors for SRCs. Statistical significance was defined as a value of *p* less than 0.05 in the aforementioned analysis.

## Results

### Clinical Characteristics and Prevalence of SRCs in Patients With T2DM

**Figure 1:**
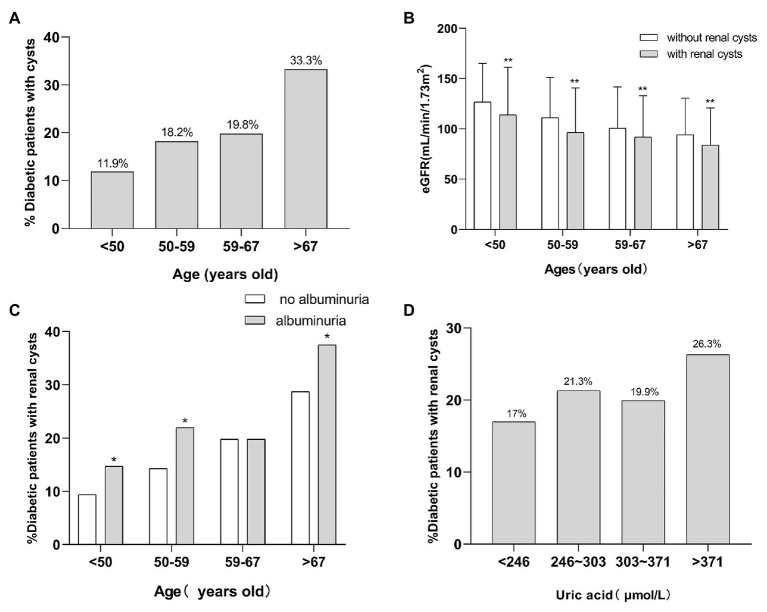
The relationship of SRCs with age, eGFR, proteinuria, and uric acid. **(A)** Incidence of SRCs in four age groups in patients with T2DM. **(B)** The eGFR of patients with T2DM with SRCs and without SRCs at different ages. **(C)** The incidence of SRCs in patients with T2DM with proteinuria and without at different ages. **(D)** The incidence of SRCs in patients with type 2 diabetes with a high level and normal level of uric acid at different ages. ^*^p < 0.05 and ^**^p < 0.01. Error bars indicate standard errors. T2DM, type 2 diabetes mellitus; SRCs, simple renal cysts; eGFR, estimated glomerular filtration rate.

Four thousand three hundred and four hospitalized patients with T2DM were analyzed. Table 1 provides the baseline characteristics of the enrolled patients. The prevalence of SRCs among the 4,304 patients was 21.1%. Patients with SRCs were older and had a higher proportion of males than patients without SRCs. These patients were divided into four groups according to age quartiles. The number of patients that ages <50, 50–59, 60–67, >67 were 956 patients, 1,182 patients, 1,050 patients, 1,116 patients, respectively. And the incidence of SRCs was 11.9, 18.2, 19.8, and 33.3% with increasing age. The value of *p* was significant (*χ*^2^ = 155.62, *p* < 0.01; Figure 1A). A higher percentage of patients with SRCs (68%) was male than patients without SRCs (56.2%). Patients with SRCs had higher SBP, longer disease course, and higher BMI and waist-hip ratio. In the clinical biochemical examination, patients without SRCs showed relatively lower hemoglobin levels, platelet count, total triglyceride, cholesterol, albumin, fasting blood glucose, and glycosylated hemoglobin levels. The median and distribution of C-peptide levels at 0 and 120 min were higher in the SRC group. Compared with patients without SRC, the SRC group had higher serum creatinine, uric acid, and 24-h urinary albumin levels and lower eGFR, calcium, and phosphorus levels (Table 1).

**Table 1:** Clinical characteristics of patients with type 2 diabetes with and without simple renal cysts (SRCs).

	Without renal cysts (*n* = 3,955)	With renal cysts (*n* = 909)	*p*
Age (years)	56.88 ± 12.04	62.87 ± 11.17	<0.01
Male (%)	1,907 (56.2%)	618 (68.0%)	<0.01
SBP (mmHg)	137.57 ± 20.88	139.72 ± 20.53	<0.01
DBP (mmHg)	81.12 ± 12.03	80.53 ± 12.25	0.19
Duration (years)	8(3, 13)	10(4, 14)	<0.01
BMI (kg/m^2^)	24.75 ± 3.64	25.04 ± 3.55	0.04
Waist-hip ratio	0.94 ± 0.07	0.95 ± 0.07	<0.01
Hemoglobin (g/L)	128.86 ± 21.01	125.64 ± 21.3	<0.01
PLT (10^9/L)	212.79 ± 74.29	205.73 ± 70.07	0.01
TG (mmol/L)	1.61(1.11, 2.47)	1.53(1.09, 2.31)	0.01
TC (mmol/L)	4.51 ± 1.27	4.36 ± 1.21	<0.01
HDL-C (mmol/L)	1.03 ± 0.32	1.02 ± 0.28	0.30
LDL-C (mmol/L)	2.83 ± 0.97	2.77 ± 1.03	0.11
ALB (g/l)	36.4 ± 4.9	35.94 ± 4.98	0.01
FPG (mmol/L)	8.49 ± 3.65	8.09 ± 3.69	<0.01
HbA1c (%)	9.09 ± 2.31	8.78 ± 2.35	<0.01
C-peptide 0 min (pmol/l)	373.9(239.3, 584.4)	437.6(268.7, 674.5)	<0.01
C-peptide 120 min (pmol/l)	826(461.1, 1,296)	954(554.5, 1501.4)	<0.01
SCr (umol/L)	82.93 ± 66.76	104.26 ± 95.99	<0.01
eGFR (ml/min/1.73 m^2^)	108.65 ± 40.93	92.38 ± 42.1	<0.01
UA (μmol/L)	311.22 ± 96.32	326.99 ± 100.78	<0.01
Urine PH	5.67 ± 0.72	5.71 ± 0.73	0.08
24 h urinary albumin (mg/d)	27.5(7.9, 16.8)	49.9(109, 296.6)	<0.01
Calcium (mmol/L)	2.18 ± 0.14	2.16 ± 0.15	<0.01
Phosphorus (mmol/L)	1.03 ± 0.21	1 ± 0.22	<0.01

Continuous variables of normal distribution were expressed as mean ± SD and compared by the Student’s *t*-test. Non-normal distribution variables were expressed as median (interquartile range 25–75%) and compared by the Mann-Whitney U test. BMI, body mass index; SBP, systolic blood pressure; DBP, diastole pressure; PLT, platelets; TG, triglyceride; TC, total cholesterol; HDL-C, high-density lipoprotein cholesterol; LDL-C, low-density lipoprotein cholesterol; ALB, albumin; FBG, fasting plasma glucose; HbA1c, glycated hemoglobin; SCr, serum creatinine; eGFR, estimated glomerular filtration rate; UA, uric acid.

In addition, regarding personal history, the number of patients with T2DM and SRCs who were smokers was larger than that of patients without SRCs. The proportion of quitters was higher among patients with SRCs than among patients without SRCs. The SRC group included patients with an overall reduced intake of alcohol compared with the non-SRC group. Among the complications of diabetes, the proportion of patients with DF, DN, and ketoacidosis was higher in the SRC group. A higher proportion of patients in the SRC group had a history of kidney stones and gout. A higher percentage of patients with T2DM and renal cysts also suffered from hypertension, CHD, cerebrovascular disease, and fatty liver. Regarding the treatments for diabetes, a higher proportion of patients with renal cysts took oral ARBs and oral hypoglycaemic drugs. Notably, the proportion of patients with renal cysts using insulin was lower. Little difference was observed in the other clinical characteristics between the two groups (Table 2).

**Table 2:** Chi-square test.

*n* (%)	Without renal cyst (*n* = 3,395)	With renal cysts (*n* = 909)	*χ*^2^	*p*
Smoking (%)	883 (26.0)	259 (28.5)	17.002	<0.01
Smoking cessation (%)	316 (9.3)	120 (13.2)		
Drinking (%)	676 (19.9)	180 (19.8)	8.071	0.02
Drinking cessation (%)	206 (6.1)	79 (8.7)		
Family history (%)	1,185 (34.9)	305 (33.6)	0.578	0.45
Urolithiasis (%)	102 (3.0)	45 (5.0)	14.007	<0.01
Postoperative (%)	74 (2.2)	32 (3.5)		
History of gout (%)	82 (2.4)	44 (4.8)	14.840	<0.01
DR (%)	1,097(32.3)	272 (29.9)	1.887	0.17
DPN (%)	1835 (54.1)	494 (54.3)	0.025	0.87
DAN (%)	133 (3.9)	29 (3.2)	1.047	0.31
DF (%)	222 (6.5)	43 (4.7)	4.059	0.04
DN (%)	1,148 (33.8)	390 (42.9)	25.798	<0.01
DK (%)	266 (7.8)	45 (5.0)	8.900	<0.01
Hypertension (%)	1,931 (56.9)	612 (67.3)	32.383	<0.01
CHD (%)	659 (19.4)	257 (28.3)	33.612	<0.01
CVD (%)	434 (12.8)	154 (16.9)	10.511	<0.01
Fatty liver (%)	1,433 (42.2)	349 (38.4)	4.302	0.04
UTI (%)	286 (8.4)	62 (6.8)	2.481	0.12
ACEIs (%)	646 (19.0)	182 (20.0)	0.456	0.50
ARBs (%)	841 (24.8)	252 (27.7)	3.296	0.07
Lipid-lowering therapy (%)	2,556 (75.3)	706 (77.7)	2.215	0.14
Oral hypoglycemic agent (%)	2,588 (76.2)	672 (73.9)	2.069	0.15
Insulin therapy (%)	2,388 (70.3)	599 (65.9)	6.663	0.01

Categorical variables were expressed as percentages and compared by the Chi-square test. Accompanied diseases and therapies during the hospital stay of subjects with and without SRC. Categorical variables were expressed as percentages and compared by the Chi-square test. DR, diabetic retinopathy; PVD, peripheral vascular; DAN, diabetic autonomic neuropathy; DF, diabetic foot; DN, diabetic nephropathy; DK, diabetic ketosis; CHD, coronary heart disease; CVD, cerebrovascular disease; UTI, urinary tract infection; ACEIs, angiotensin converting enzyme inhibitors; ARBs, angiotensin receptor blockers.

### Decreased Renal Function in Patients With T2DM Complicated With SRCs

**Figure 2:**
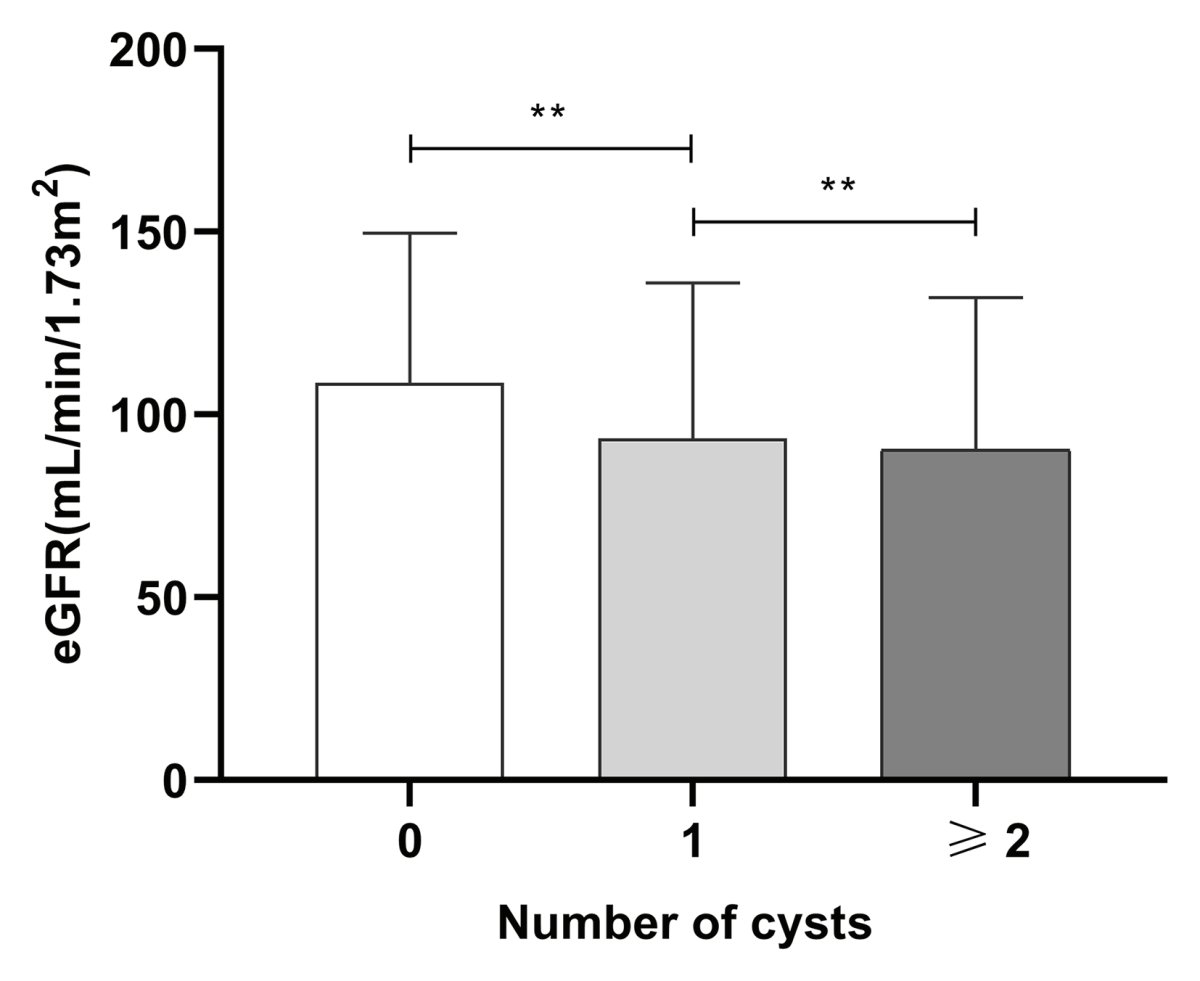
The relationship between numbers of SRCs and eGFR in patients with type 2 diabetes. Mean ± standard deviation: no SRCs group: 108 ± 40.93 ml/min/1.73 m^2^, one SRC: 93.53 ± 42.5 ml/min/1.73 m^2^, and more than two SRCs: 90.34 ± 41.72 ml/min/1.73 m^2^. SRC, simple renal cysts; eGFR, estimated glomerular filtration rate; ^**^p < 0.01. Error bars indicate standard errors.

The distribution of renal cysts is shown in Supplementary Table 1. The exact location and number of cysts in 20 patients with cysts were missing on account of the long range of time of the study and negligence of the sonographer. Thus, they were excluded in the analysis of the exact number and location of cysts. The number of patients with multiple cysts was 477 (52.6%) and the number of patients with single cysts was 412 (45.3%). Most of the patients with SRCs had multiple renal cysts, and 252 patients had bilateral renal cysts (28.35%). Patients were divided into three groups according to the number of renal cysts: no renal cysts, single renal cyst, and multiple renal cysts (number ≥ 2). According to the results of one-way ANOVA, the eGFR of the three groups satisfied the homogeneity of variance assumption, and the eGFR decreased as the number of SRCs increased (*p* < 0.05; Figure 2). Univariable analysis showed that the SRCs were associated with decreased eGFR which was defined as less than or equal to 124 ml/min/1.73 m^2^ [OR = 1.99, 95% confidence interval (1.7, 2.3), *p* < 0.01]. After adjusting for the confounders, such as males, age > 59 years old, duration > 10 years, urolithiasis, hemoglobin < 110 g/l, ALB < 40 g/l, TG > 1.71 mmol/l, HbA1c > 6%, UA > 416 (male) or 357(female; μmol/L), MAAU, DAN, DN, DK, hypertension, CHD, CVD, UTI, phosphorus > 1.51 mmol/L, the association between SRCs and decreased eGFR was still significant [odds ratio = 1.49, 95% confidence interval (1.24, 1.79), *p* < 0.01; Table 3]. The size of the SRCs was measured by the cross-sectional area of the largest renal cyst [*S* = the length (mm) × width of the largest renal cyst (mm)]. Patients were divided into two groups based on the median value, *S* < 224 mm^2^ and *S* ≥ 224 mm^2^, to further study the effect of the size of renal cysts on the renal function of patients. The distribution of the SRC based on the size is shown in the Supplementary Figure 1. However, the Wilcoxon rank-sum test showed no significant difference in eGFR between the two groups. The distribution of renal lengths was approximately the same in the two groups, suggesting that the kidney lengths in patients with cysts were unable to be classified as a subpopulation of particularly small kidneys (Supplementary Figure 2). The mean combined length of the right and left kidneys was smaller in patients with SRCs (20.8 ± 1.58 cm) than in patients without SRCs (20.9 ± 1.56 cm, *p* = 0.043). However, the statistical significance might be due to the large population. Moreover, when patients were classified according to unilateral, bilateral, single, multiple, and no renal cysts, no significant differences were observed in the length of the kidney between the two groups (Supplementary Figure 3).

**Table 3:** The univariable and multivariables logistic regression analysis of eGFR in patients with type 2 diabetes mellitus (T2DM).

Variables	Unadjusted OR (95% CI)	*p*	Adjusted OR (95% CI)	*p*
SRCs	1.99 (1.7, 2.3)	<0.01	1.49 (1.24, 1.79)	<0.01

Adjusted confounders variables included gender, age, duration, smoking, drinking, BMI, family history, urolithiasis, hemoglobin, TC, TG, LDL, ALB, HbA1c, UA, 24 h urinary albumin, calcium, phosphorus, DR, DPN, DAN, DF, DN, DK, hypertension, CVD, CHD, fatty liver, UTI, ACEIs, ARBs, lipid-lowering therapy, oral hypoglycemic agent, insulin therapy, and SRCs. The eGFR were divided into two group, eGFR > 105.21 ml/min/1.73 m^2^ and eGFR ≤ 105.21 ml/min/1.73 m^2^. OR, odds ratio; CI, confidence interval; eGFR, estimated glomerular filtration rate; T2DM, type 2 diabetes; SRCs, simple renal cysts; HbA1c, glycated hemoglobin; UA, uric acid; DR, diabetic retinopathy; PVD, peripheral vascular; DAN, diabetic autonomic neuropathy; DF, diabetic foot; DN, diabetic nephropathy; DK, diabetic ketosis; CHD, coronary heart disease; CVD, cerebrovascular disease; UTI, urinary tract infection; ACEIs, angiotensin converting enzyme inhibitors; ARBs, angiotensin receptor blockers.

### SRCs Correlated With Age, Gout, Proteinuria, and CVD in Patients With T2DM

**Figure 3:**
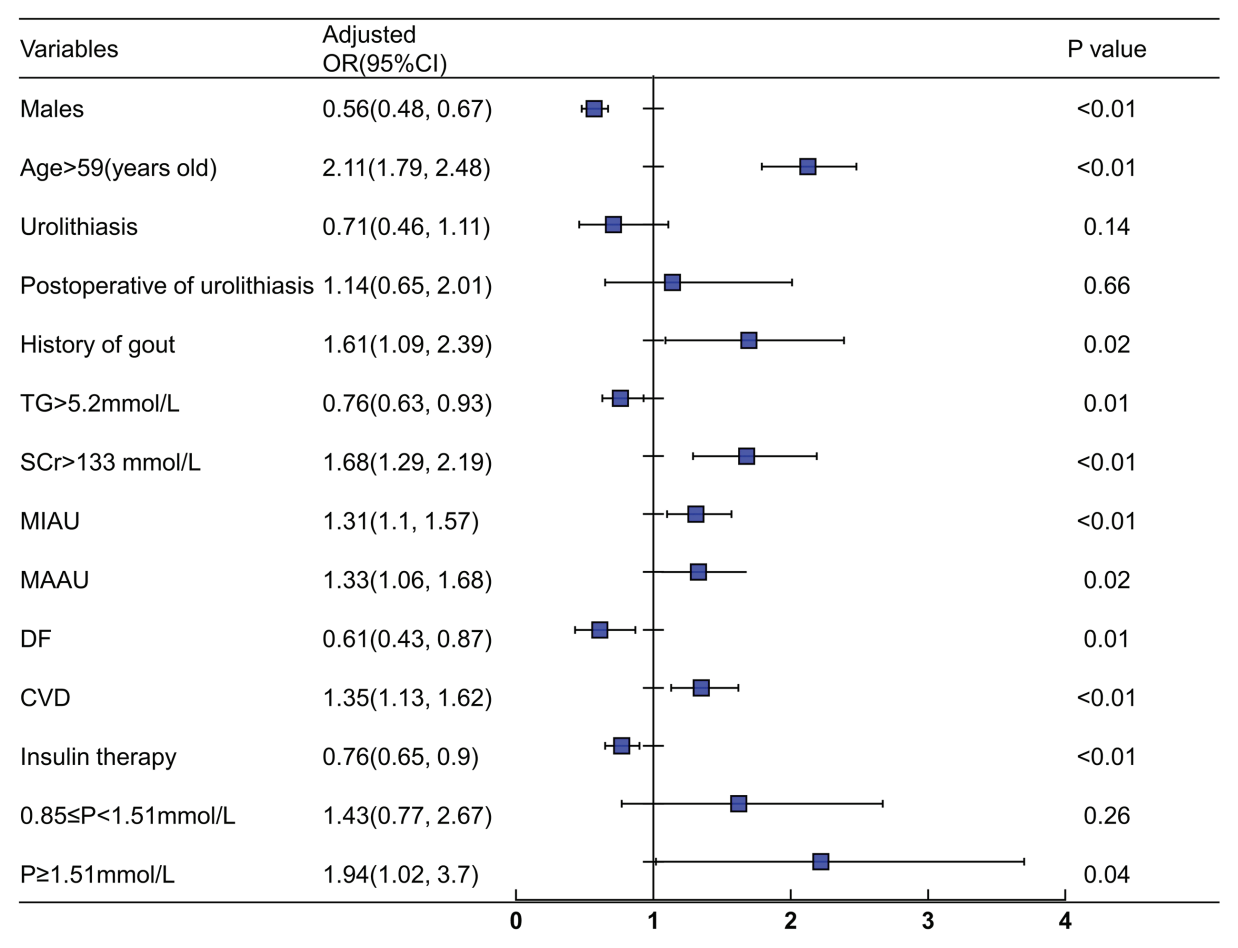
Logistic regression analysis for simple renal cysts. Adjusted confounders variables included gender, age, duration, smoking, drinking, urolithiasis, waist-hip ratio, HB, PLT, TG, Scr, ALB, FPG, HbA1c, 24 h urinary albumin, Ca, P, DK, DF, hypertension, CVD, fatty liver, UTI, CVD, and insulin therapy. SRCs, simple renal cysts; SCr, serum creatinine; TG, triglyceride; CVD, cerebrovascular disease; DF, diabetic foot; MIAU, microalbuminuria (30–300 mg/24 h); MAAU, microalbuminuria (>300 mg/24 h); DF, diabetic foot.

Variables that were significantly different in patients with and without SRCs were included in the logistic analysis (except for SBP, BMI, 0 min C-peptide level, 120 min C-peptide level, UA, and DN), including age, gender, disease duration, waist-hip ratio, hemoglobin, PLT, TG, TC, FPG, HbA1c, SCr, 24 h urinary albumin, calcium, phosphorus, smoking, drinking, urolithiasis, history of gout, DF, DK, hypertension, CVD, fatty liver, and insulin therapy (Tables 1 and 2). A multivariate logistic regression analysis was conducted to identify the risk factors for SRCs, and age, gout, proteinuria, CVD, and increased serum phosphorus levels were independent risk factors for SRCs in patients with T2DM. Urolithiasis, postoperative urolithiasis, and normal or decreased phosphorus levels were not significant risk factors for SRCs (Figure 3).

### Poor Renal Function and Uric Acid Levels Were Associated With SRCs in Patients With T2DM Stratified by Age

Since age strongly correlated with SRCs, the association between age and SRCs was further analyzed. The patients were divided into four groups according to age quartiles. According to the ANOVA, the eGFR of patients in the four groups satisfied the homogeneity of variance assumption, and the decrease in the eGFR of the SRC group was more significant than that in the group without SRCs (*p* < 0.05; Figure 1B). Regarding proteinuria, except for the group in which the ages ranged from 59 to 67 years old, the incidence of SRCs increased significantly in patients with proteinuria in the different age groups (*p* < 0.05; Figure 1C). Patients were divided into four groups according to the quartiles of uric acid levels. As shown in Figure 1D, the prevalence of SRCs significantly increased as the uric acid levels increased (*p* < 0.05). In addition, among patients over 67 years old, the incidence of SRCs was significantly increased in patients with a high level of uric acid compared to patients with normal level of uric acid, but a noticeable difference was not observed in other age groups (Supplementary Figure 4).

## Discussion

The incidence of SRCs increases with age, ranging from 3 to 41% in groups stratified by age in 10 year intervals (Terada et al., 2004; Chang et al., 2007; Mosharafa, 2008; Choi, 2016). Most researchers agree that SRCs are generally asymptomatic and have little influence on renal function (Ozdemir and Kapucu, 2017). However, patients with SRCs were reported to have smaller renal volumes and a lower eGFR than patients without SRCs, suggesting that isolated cysts might be an early manifestation of renal unit loss (Al-Said and O’Neill, 2003). Subsequently, they further showed that the presence of renal cysts detected by CT was correlated with age, gender, the creatinine clearance rate, and eGFR in hospitalized patients (Al-Said et al., 2004). The risk of chronic kidney failure in patients with renal cysts is 1.5 times higher than that in patients without renal cysts (Al-Said and O’Neill, 2003). Studies in the Chinese population show that participants with SRC were more susceptible to proteinuria and renal insufficiency and that the maximum diameter of the cyst contributed to the decline in renal function (Kong et al., 2018; Chen et al., 2019). Based on these data, SRCs are common renal diseases that potentially result in a decrease in renal function.

On the other hand, in patients with T2DM, the incidence of SRCs in Chinese males and postmenopausal females is 18.1% (Han et al., 2017). However, the relationship between SRCs and renal function in patients with T2DM remains unclear. Here, the incidence of SRCs was 21.1% in patients with T2DM. The incidence of SRCs was up to 33.3% in patients aged greater than 67 years. This result is consistent with the findings from previous studies showing that diabetic status increases the incidence of renal cysts (Ozveren et al., 2016). The potential explanation for the difference in the incidence of SRCs compared with healthy subjects is differences in ethnicity, environmental exposure, and distributions of age and gender.

The risk factors for SRCs in healthy subjects are age, hypertension, and chronic renal insufficiency (Terada et al., 2004; Chang et al., 2007). In addition, Hasegawa et al. (2013) reported an increased prevalence of SRCs in patients with gout, indicating that gout might be related to the formation of SRCs. For patients with T2DM, gender, age, hyperuricaemia, proteinuria, and hyperuricaemia excretion score are independent risk factors for renal cysts (Han et al., 2017). However, in addition to these factors, CVD, an increased serum phosphorus level, and reduced renal function were identified to be associated with SRCs in the present study.

With regard to uric acid and SRCs, we observed the incidence of SRCs in patients stratified by different uric acid levels to further investigate the association between SRCs and gout. When the uric acid level exceeded 371 μmol/L, the prevalence of SRCs was remarkably increased compared to patients with a lower level of uric acid. In addition, in contrast to the results from other studies, gender was not related to SRCs in our study. This difference might be attributed to the use of different inclusion criteria. In the present study, all subjects were enrolled, regardless of gender. Hypertension was previously identified as a risk factor for SRCs in healthy subjects. However, our results are consistent with a study that reported no association between hypertension and SRCs in diabetic patients (Han et al., 2017). In the diabetic population, the relationship between hypertension and SRCs was not significant. The reason may be the difference in the study population, sample size, stage of hypertension, and techniques for detecting renal cysts (Lee et al., 2013; Choi, 2016; Mensel et al., 2018). However, the potential explanations for the difference must be clarified by conducting further research.

This study is the first to show that CVD and an increased serum phosphorus level positively correlated with SRCs. According to Wu et al. (2019), the presence of SRCs is associated with increased arterial stiffness, which might explain the correlation of SRCs and CVD in T2DM. The mechanisms might include abnormal arginine vasopressin (AVP) levels and a low resistance of the renal circulation. Additionally, since MMP-2 and MMP-9 have been detected in both benign and malignant renal cysts (Harada et al., 2002) and the abnormal expression of MMP-2 and MMP-9 are pertinent to the pathogenesis of atherosclerosis the altered expression and distribution of MMPs might contribute to the correlation between SRCs and CVD (Wagsater et al., 2011). Furthermore, hyperphosphatemia contributes to higher cardiovascular and all-cause mortality rates among patients with chronic kidney disease (CKD; Da et al., 2015; Jeon et al., 2017). The potential underlying mechanism is aortic and coronary artery calcification and endothelial dysfunction caused by an increased serum phosphorus level (Shuto et al., 2009). Additionally, hyperphosphatemia stimulates vascular smooth muscle cells to express osteogenic markers and predisposes them to calcification (Giachelli et al., 2001). These changes might be possible explanations for the relationship between hyperphosphatemia and SRCs in patients with T2DM.

The next issue was the relationship between SRCs and renal function. SRCs negatively correlated with the eGFR in patients with T2DM, consistent with the results obtained from healthy subjects (Al-Said and O’Neill, 2003; Al-Said et al., 2004). Logistic analysis showed that the SRC was associated with eGFR > 105.21 ml/min/1.73 m^2^, even after adjusting for confounders. A study in a healthy population with follow up data indicated that participants with SRC had a higher incidence of proteinuria and renal insufficiency, which was similar to our results (Chen et al., 2019). Due to age and renal function are closely related (O’Sullivan et al., 2017), we analyzed the renal function and SRCs after stratifying patients by age to address the potential confounding effect of age. Furthermore, most young patients have less duration of diabetes and renal function were relatively better than other groups. Thus, the average eGFR when patients less than 50 years were higher than other age groups. A more significant reduction in the eGFR was observed in the SRC group than in patients without SRCs in different age groups. In addition, the number of SRCs negatively correlated with the eGFR in patients with T2DM, while the size of SRCs was independent of the eGFR in patients with T2DM. Thus, SRCs were likely related to the decrease in renal function in patients with T2DM.

On the other hand, the kidney size of patients with multiple cysts is slightly larger in the clinic. This study found that the combined kidney length of patients with renal cysts was slightly smaller than that of patients without SRCs. This slight difference might be due to the large number of patients (total of 4,304 patients). Therefore, the difference of kidney size between the two groups was negligible for diabetic patients in this study. Regarding the large volume of kidneys with multiple cysts, we also found this problem in clinical practice, but there was no obvious difference in this study between the two groups. The reason may be the large sample size of the study, and the average value of eGFR in our study population is more than 90 ml/min/1.73 m2, which reflects that there is no significant relationship with CKD. Therefore, this study did not reflect the size of the kidney changes but it is certainly worth further study on this subject. Furthermore, the number and location of renal cysts were not correlated with the renal length in the comparison between the two groups.

## Conclusion

In summary, we observed a higher incidence of SRCs in patients with T2DM. The SRC was correlated with proteinuria and reduced eGFR in patients with T2DM. More attention should be paid to gout, proteinuria, CVD, serum phosphorus levels, and renal function in T2DM patients with SRCs.

## Data Availability Statement

The original contributions presented in the study are included in the article/Supplementary Material, and further inquiries can be directed to the corresponding author.

## Ethics Statement

The studies involving human participants were reviewed and approved by The Medical Ethics Committee of the Second Xiangya Hospital of Central South University. The ethics committee waived the requirement of written informed consent for participation.

## Author Contributions

LS: conception and design. LW, YX, and XX: collection and assembly of data. LW, LL, YH, HZ, and MY: data analysis and interpretation. LW: manuscript writing. YY: read through and corrected the manuscript. All authors contributed to the article and approved the submitted version.

### Conflict of Interest

The authors declare that the research was conducted in the absence of any commercial or financial relationships that could be construed as a potential conflict of interest.
