# Supplementary material for: The Relationship Between Simple Renal Cysts and Renal Function in Patients With Type 2 Diabetes
Source: Front Physiol. 2020 Dec 15;11:616167. doi: 10.3389/fphys.2020.616167 (PMC7770177; doi:10.3389/fphys.2020.616167)
Supplement: Supplementary file 1 [file Image_1.pdf]

Supplementary Material

1 Supplementary Figures and Tables

1.1 Supplementary Figures

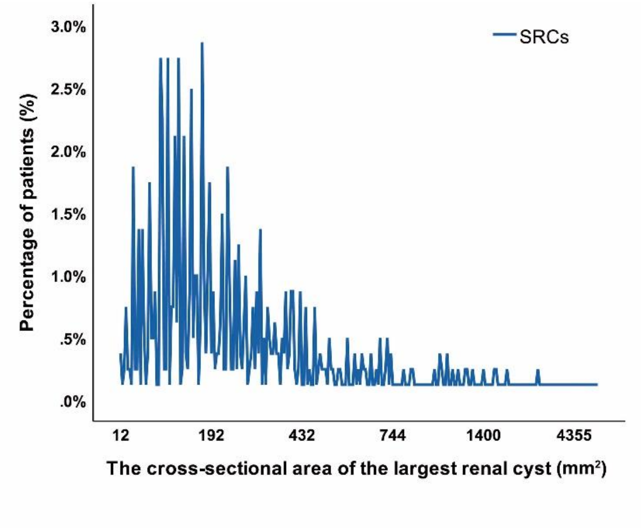

Supplementary Figure 1. The distribution of simple renal cysts group based on size in patients with type 2 diabetes

The size of the SRCs was measured by the cross-sectional area of the largest renal cyst (S = the length (mm) × width of the largest renal cyst (mm)).

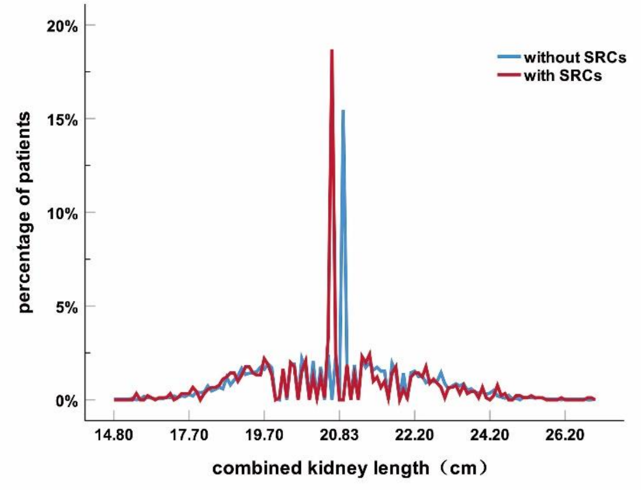

Supplementary Figure 2. The distribution of combined kidney lengths in the SRCs group and non SRCs group

Combined kidney length means the sum of the left and right kidney length. SRCs, simple renal cysts

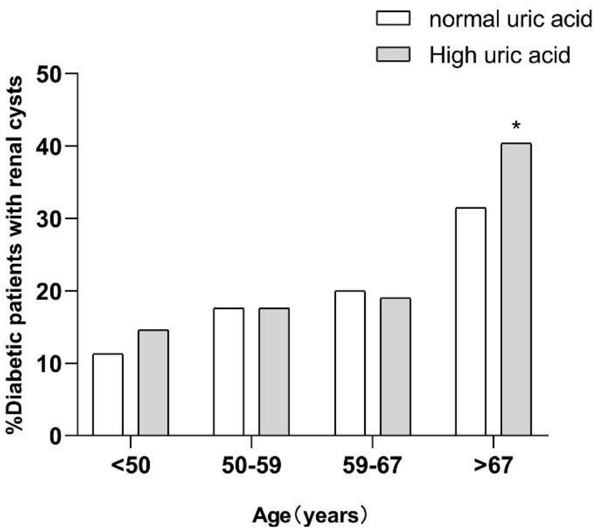

Supplementary Figure 3. Relationship of the kidney length, numbers and distribution of simple renal cysts.

Error bars indicate standard errors.

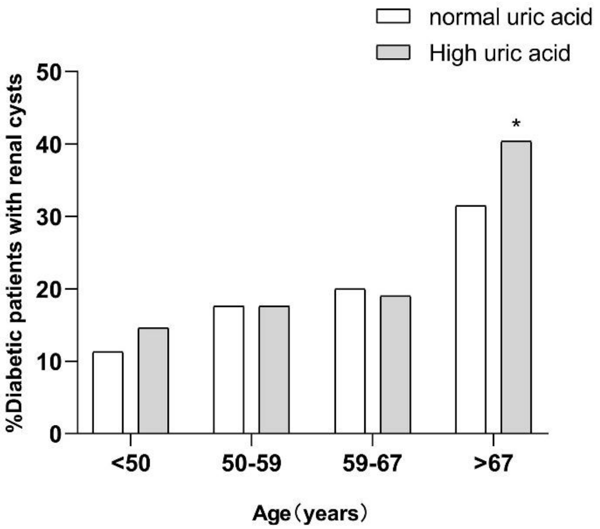

Supplementary Figure 4.The incidence of SRCs in type 2 diabetic patients with high levels and normal levels of uric acid at different ages

* $P<0.05$ vs. normal uric acid. Error bars indicate standard errors. SRCs, simple renal cysts

1.2 Supplementary Tables

Supplementary-Table 1 Distribution of SRCs in type 2 diabetic patients

	Total numbers of cysts	
	1	≥2
Left kidney	272(29.9%)	168(18.5%)
Right kidney	140(15.4%)	57(6.3%)
Bilateral	0(0%)	253(27.8%)
Total	412(45.3%)	477(52.6%)

Supplementary-Table 1 Numbers of patients are given in the table and the percentage was presented in parentheses. SRC, simple renal cyst.
